# Thyroid Lobectomy Is Associated with Excellent Clinical Outcomes in Properly Selected Differentiated Thyroid Cancer Patients with Primary Tumors Greater Than 1 cm

**DOI:** 10.1155/2013/398194

**Published:** 2013-12-23

**Authors:** Fernanda Vaisman, Denise Momesso, Daniel A. Bulzico, Cencita H. C. N. Pessoa, Manuel Domingos Gonçalves da Cruz, Fernando Dias, Rossana Corbo, Mario Vaisman, R. Michael Tuttle

**Affiliations:** ^1^Endocrinology Service, Universidade Federal do Rio de Janeiro, Rua Rodolpho Paulo Rocco 255, Cidade Universitária Ilha do Fundão, 21941-913 Rio de Janeiro, RJ, Brazil; ^2^Endocrinology Service, Instituto Nacional do Cancer, Praça da Cruz Vermelha 23, Centro, 20230-130 Rio de Janeiro, RJ, Brazil; ^3^Surgery Service, Universidade Federal do Rio de Janeiro, Rua Rodolpho Paulo Rocco 255, Cidade Universitária Ilha do Fundão, 21941-913 Rio de Janeiro, RJ, Brazil; ^4^Head and Neck Service, Instituto Nacional do Cancer, Praça da Cruz Vermelha 23, Centro, 20230-130 Rio de Janeiro, RJ, Brazil; ^5^Endocrinology Service, Memorial Sloan Kettering Cancer Center, 1275 York Avenue, New York, NY 10065, USA

## Abstract

*Background and Objective*. An individualized risk-based approach to the treatment of thyroid cancer is being extensively discussed in the recent literature. However, controversies about the ideal surgical approach remain an important issue with regard to the impact on prognosis and follow-up strategies. This study was designed to describe clinical outcomes in a cohort of low and intermediate risk thyroid cancer patients treated with thyroid lobectomy. *Methods*. Retrospective review of 70 patients who underwent lobectomy. *Results*. After a median follow-up of 11 years, 5 patients (5/70, 7.1%) recurred and 5 had a completion for benign lesions, while 60 patients (86%) continued to be observed without evidence for disease recurrence. Suspicious ultrasound findings were significantly more common in patients that had structural disease recurrence (100% versus 4.3%, *P* < 0.001). Furthermore, a rising suppressed Tg value over time was also associated with structural disease recurrence (80% versus 21.5%, *P* = 0.01). After additional therapy, 99% of the patients had no evidence of disease. *Conclusions*. Properly selected thyroid cancer patients can be treated with lobectomy with excellent clinical outcomes.

## 1. Introduction

While most of the clinicians advocate total thyroidectomy for nearly all patients with differentiated thyroid cancer (DTC), there are also those who prefer a more individualized surgical approach based on personalized estimates of risk of recurrence and risk of disease specific mortality. The prognosis of DTC is generally good, especially for young patients with small tumors. Hay et al. [1] from the Mayo Clinic and Shaha et al. [2] from the Memorial Sloan-Kettering Cancer Center have reported 20-year disease specific survival rates of 99% in low-risk patients. In several major centers, total thyroidectomy is performed for aggressive tumors, while hemithyroidectomy is offered to properly selected patients who have small tumors without extrathyroidal spread or metastases [3–5].

Most of the current guidelines recommend total thyroidectomy as the initial surgical procedure for known papillary thyroid cancer if the primary tumor is greater than 1 cm unless there is a contraindication to this surgery [6–8]. Thyroid lobectomy is considered sufficient if the primary tumor is less than 1 cm and intrathyroidal in the absence of known metastatic disease or prior radiation history [8]. However, the National Comprehensive Cancer Network (NCCN) guidelines allow for a thyroid lobectomy as the initial surgical procedure in tumors up to 4 cm in diameter provided the patients are between 15 and 45 years of age and there is no history of prior radiation or evidence of distant metastasis, cervical lymph node metastases, or aggressive histologic variants [9].

Using a risk-based approach to the extent of initial surgery, we have previously published a structural disease recurrence rate of 4.2% at Memorial Sloan-Kettering Cancer Center in a cohort of 72 patients followed for a median of 5 years with thyroglobulin values and serial neck ultrasound, examinations after thyroid lobectomy for papillary thyroid cancer [10]. Importantly, 88% of the patients were subsequently rendered free of disease after identification and treatment of their recurrent disease. While these data were very promising, they represented a relatively small cohort, followed for a short duration at a single center with extensive experience in risk adapted thyroid cancer management.

Obviously, the decision to proceed with less than a total thyroidectomy as the initial surgical procedure has important implications with regard to follow-up recommendations. In this setting, follow-up radioactive iodine scanning is usually not helpful and serum thyroglobulin values are thought to have less value than in patients treated with total thyroidectomy and RAI ablation [11–14]. However, since the majority of recurrences in patients properly selected for less than total thyroidectomy will occur either in the contralateral lobe or in cervical lymph node metastases, neck ultrasonography remains one of the primary modalities to identify disease recurrence.

In this study, we expand our previous observations to include two additional medical centers (Hospital Universitario Clementino Fraga Filho-UFRJ and Instituto Nacional do Cancer do Rio de Janeiro) in a different country (Brazil) with a cohort of differentiated thyroid cancer patients followed for more than twice as long after initial therapy with less than total thyroidectomy. Furthermore, we carefully evaluated changes in serum thyroglobulin and the role of neck ultrasound in the detection of structural disease recurrence in these low-risk patients in order to develop a rationale approach to follow-up. Finally, clinical status after treatment of disease recurrence was evaluated to determine the effect of salvage therapy in those few patients that demonstrated structural disease recurrence during follow-up.

## 2. Subjects and Methods

### 2.1. Subjects

After obtaining IRB approval, we retrospectively reviewed the electronic medical records of 70 consecutive patients with differentiated thyroid cancer evaluated at Hospital Universitario Clementino Fraga Filho-UFRJ and Instituto Nacional do Cancer do Rio de Janeiro who had subtotal thyroidectomy. Patients less than 18 years old at diagnosis or with a histological diagnosis of medullary thyroid cancer or anaplastic thyroid cancer were excluded from the study. Patients with positive anti-thyroglobulin antibodies were also excluded from the final analysis.

The initial thyroid surgery was ipsilateral lobectomy with or without isthmusectomy. It has not been our institutional practice to perform prophylactic neck dissections in differentiated thyroid cancer.

### 2.2. Risk Stratification

Each patient (*n* = 70) was risk-stratified using the 7th edition of the AJCC/UICC staging system (Stage I, II, III, or IV) and the 2009 ATA risk of recurrence stratification system (low, intermediate, or high risk of recurrence).

### 2.3. Laboratory Studies

Between 1986 and 1997, a variety of Tg assays were used with functional sensitivities of approximately 1 *μ*g/L. From 1998 through 2000, the functional sensitivity of the Tg assay was 0.5 *μ*g/L. Since 2001, serum Tg was quantified by immunometric assay (Immulite â) with functional sensitivity of 0.2 *μ*g/L.

### 2.4. Follow-Up

Patients were usually followed every 6 months during the first year and then at 6–12-month intervals. For the past 10 years, nonstimulated serum Tg measurements and neck ultrasonography were routinely used in the follow-up of thyroid cancer patients at the discretion of the attending physician. Levothyroxine therapy to achieve a TSH goal of 0.1–0.3 mUI/L was routinely prescribed following surgery, even in these patients treated with less than total thyroidectomy.

### 2.5. Clinical Endpoints

The primary endpoint of the study was the identification of structurally evident persistent/recurrent disease during follow-up. Because serum levels of Tg cannot be used to definitively diagnosis recurrent/persistent disease in patients treated with less than total thyroidectomy and RAI, Tg levels were not used in the definition of recurrent/persistent disease.

Patients were considered to have structural evidence of recurrent disease if any of the following conditions were met: (1) positive cytology/histology or (2) highly suspicious lymph nodes or thyroid bed nodules on the neck ultrasound (hypervascularity, cystic areas, heterogeneous content, rounded shape, and enlargement on the follow-up).

Patients were considered to have no structural evidence of disease (NSED) at final follow-up if they had no evidence of structural persistent or recurrent disease confirmed by biopsy (cytology or histology) and a normal ultrasound.

Secondary endpoints included the absolute values of suppressed serum Tg levels, the trend in serum Tg levels over time, the need for additional therapy during follow-up (additional surgery and/or radioactive iodine treatment at some point during follow-up), and disease specific mortality. All patients included in the study had TSH levels <0.5 mUI/L at the time of Tg determinations during follow-up. Suppressed Tg values that increased or decreased more than 20% using the same assay in two consecutive measurements, at least 1 month apart, were considered to represent a clinically significant change.

### 2.6. Statistical Methods

Continuous data are presented as the median with minimum and maximum values provided. For comparing means, the *t*-test was used when variables had normal distribution and Mann-Whitney for nonparametric variables. For categorical data, we used Chi^2^ and Fisher exact tests. Analysis was performed using SPSS software (Version 19.0.1: SPSS Inc., Chicago, IL).

## 3. Results

Seventy patients met the inclusion criteria and had all the data necessary for analysis. As shown in Table 1, the median age at diagnosis was 35.5 years (ranging from 20 to 73 yrs) with a median primary tumor size of 2.0 cm (range 0.2–5.0 cm). Only 23% were microcarcinomas (T1a), while the remainder were T1b (37%), T2 (23%), or T3 lesions (17%). Prior to initial surgery, 46 patients had fine needle aspiration biopsy (FNA) that showed papillary thyroid cancer, 14 had inconclusive FNA, and 10 underwent surgery without FNA due to compressive symptoms and/or esthetic reasons. The majority of the patients were females (94.3%), TNM stage 1 (84.3%), and ATA low risk (70%).

**Figure 1:**
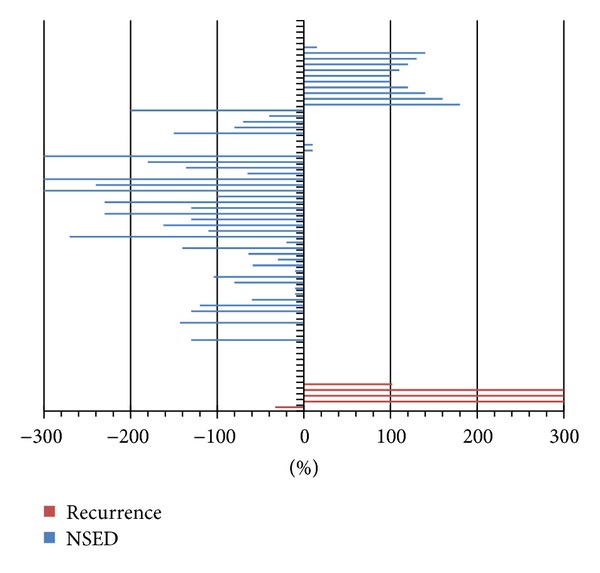
Tg variation over time. NSED: no structural evidence of disease. Variations under 20% were considered stable, *P* = 0.01.

After a median follow-up of 11 years (range of 3–24 yrs), 10 patients (14%) had a completion thyroidectomy, while 60 patients (86%) continued to be followed with observation without evidence for recurrent disease (see Figure 1). Of the 10 patients that underwent completion thyroidectomy, 5 patients (5/70, 7.1%) were found to have papillary thyroid cancer either in the contralateral lobe (3/70, 4.2%) or cervical lymph nodes (*n* = 2/70, 2.8%). The remaining 5 patients had a completion thyroidectomy for histologically proven benign findings on the basis of either suspicious nodules in the contralateral lobe with inconclusive FNA (3/70, 7.1%) or concern over a rising Tg value (2/70, 2.8%).

Routine neck ultrasonography identified the structural disease in each of the 5 patients with histologically proven disease recurrence (see Table 2). However, a comparison of the clinicopathological features of the 5 patients that had structural disease recurrence with the 65 patients that did not have disease recurrence showed no significant differences in age at presentation, gender, size of the primary tumor, histology of the primary tumor, TNM stage, or ATA risk level. Not surprisingly, suspicious ultrasound findings were significantly more common in patients that had structural disease recurrence than in those that continued to be NSED (100% versus 4.3%, *P* < 0.001). Furthermore, a rising suppressed Tg value over time (with corresponding TSH < 0.5 mUI/L) was also significantly more like to be found in patients with structural disease recurrence than in those that continued to be NSED (80% versus 21.5%, *P* = 0.01). In those patients with rising Tg and negative ultrasound, further tests, such as neck and chest CT with contrast, were not able to identify the source of the thyroglobulin. A rising Tg had a sensitivity of 80%, a specificity of 80%, and a negative predictive value of 98%. The positive predictive value was only 22% since rising Tg values were also seen in 14 patients without evidence of disease recurrence. As shown in Figure 1, the magnitude of the rise in serum thyroglobulin was significantly higher in the patients that had disease recurrence than in the patients that continued to be NSED, although the rises in Tg in 2 of the 5 recurrences were of the same magnitude as the rises seen in patients who continue to be NSED.

In addition to completion thyroidectomy and therapeutic neck dissection when indicated, 4 of the 5 patients with histologically proven disease recurrence subsequently underwent radioactive iodine remnant ablation (each demonstrating uptake only in the thyroid bed on posttherapy scan). At the time of final follow-up, all patients were still alive, with 69/70 patients (99%) classified as having no evidence of disease. No patients developed distant metastases. Salvage therapy was very effective with 4/5 patients that had histologically confirmed disease recurrence being rendered no evidence of disease with completion thyroidectomy with or without RAI ablation (followed for an additional 7–24 yrs after disease recurrence). One patient (1/70, 1%) has persistent disease manifest by a 8 mm level II metastatic lymph node being followed with active surveillance.

## 4. Discussion

This study provides further evidence that thyroid lobectomy is very effective primary therapy for properly selected patients with differentiated thyroid cancer. Nearly 80% of this cohort had a primary tumor greater than 1 cm and, despite that, experienced 100% disease specific survival over a median of 12 years with a structural disease recurrence rate of only 7.1%. Furthermore, additional therapy provided at the time of structural disease recurrence rendered 80% of the patients disease-free. Overall, 99% of the patients were disease-free at final follow-up. Even if all patients had received total thyroidectomy and RAI ablation, it is hard to imagine better clinical outcomes at final follow-up. These data also indicate that this selective use approach to lobectomy can be effectively implemented in multiple centers in different countries and is not unique to the experience of a single cancer center.

The structural disease recurrence rate in this study (7.1%) was slightly higher than in our previous report (4.2%), maybe due to the small number of patients in this group and all the recurrences occurred in the first two years indicating that this was present since the beginning and for some reason was not detected in the first evaluation. As expected, neck ultrasound during follow-up was the primary modality effective in identifying structural disease recurrence in both this study and our previous study.

In the current study, a rising serum Tg was seen more frequently in patients that had structural disease recurrence than in those that did not (80% versus 22%, *P* < 0.01). However, the clinical utility of changes in serum Tg is limited by the observation that a rising Tg was also seen in 20% of patients that were classified as having no evidence of disease.

This resulted in a PPV of only 22% indicating that a rising Tg value is not very specific for disease recurrence. However, the NPV was 98% indicating that the lack of a rise in serum Tg is a good clinical sign associated with a low risk of structural disease recurrence. Previous studies showed that patients who underwent total thyroidectomy and RAA but had persistent detectable serum Tg after initial therapy that declines spontaneously over time can be used as a marker of good prognosis [15–17]. In contrast, a rising of serum Tg over time is well accepted as a marker of high risk for recurrence in patients treated with total thyroidectomy and RAI ablation [8, 16]. Our data confirm previous observations that indicate that increases in serum Tg over time are less specific for disease recurrence in patients treated with lobectomy compared with patients receiving total thyroidectomy and RAI remnant ablation. Despite being a relatively small study, the findings are intriguing and suggest that Tg values may be useful in patients that did not receive RAI ablation.

A very important finding of this study is the effectiveness of salvage therapy utilized to treat the few disease recurrences identified. Additional therapy (surgery and/or RAI) effectively removed all of the diseases in 80% of the patients. Each of these patients has subsequently been followed for 7–24 years after additional therapy without evidence for persistent or recurrent disease. Only one patient developed a newly identified small lymph node metastasis during follow-up.

In conclusion, we report excellent clinical outcomes in properly selected differentiated thyroid cancer patients treated with thyroid lobectomy as primary therapy and followed with serial neck ultrasound and thyroglobulin evaluations. The primary modality for detection of structural disease recurrence was the neck ultrasound, while a trend in serum Tg over time that was stable or declining made it very unlikely that disease recurrence would be identified. Therefore, consistent with the NCCN guidelines, we consider thyroid lobectomy to be an acceptable alternative to total thyroidectomy in properly selected differentiated thyroid cancer patients with primary tumors greater than 1 cm.

## Figures and Tables

**Table 1:** Characteristics of the entire cohort.

	*N* = 70	%
Age	35,5 (20–73)	—
Gender (F/M)	66/4	94.3/5.7
Size	2.0 (0.2–6.0)	—
Histology		
Papillary	56	80
Follicular	14	20
Unifocal	59	84.3
Multifocal	11	15.7
T		
T1a	16	22.9
T1b	26	37.1
T2	16	22.9
T3	12	17.1
T4	0	
N		
Nx	44	62.9
N0	14	20
N1a	5	7.1
M0	0	
TNM		
Stage I	59	84.3
Stage II	7	5.7
Stage III	4	10
Stage IV	0	
ATA		
Low	49	70
Intermediate	21	30
High	0	0
Follow-up	12 (3–28)	—
Additional therapy	10	14.3
Time to additional therapy (years)	11 (3–24)	—
First suppressed Tg post-op	4.23 (<0.5–46)	—
Trend of Tg		
Decrease	29	41.4
Increase	18	25.7
Stable	23	32.9
Final status		
NSED without additional therapy	60	85.7
NSED with additional therapy	9	12.9
Recurrent/persistent disease	1	1.4

**Table 2:** Patients that had recurrent disease.

	Patient 1	Patient 2	Patient 3	Patient 4	Patient 5
Gender	F	F	F	F	F
Age	37	57	46	54	21
Tumor size	3,0 cm	2,0 cm	1,0 cm	2,5 cm	0,8 cm
ATA Stage	Intermediate	Low	Intermediate	Low	Low
Histology	Papillary	Papillary	Papillary	Papillary	Papillary
TG trend	Decreasing	Increasing	Increasing	Increasing	Increasing
Time from primary treatment to recurrence	13 months	6 months	6 months	9 months	8 months
Location of recurrence	Central neck lymph node	Contralateral lobe	Contralateral lobe	Contralateral lobe	Contralateral lobe
